# Levels of Platinum Group Metals in Selected Species (*Sarotherodon melanotheron*, *Chonophorus lateristriga*, *Macrobrachium vollenhovenii* and *Crassostrea tulipa*) in Some Estuaries and Lagoons Along the Coast of Ghana

**DOI:** 10.1100/tsw.2010.197

**Published:** 2010-10-12

**Authors:** D. K. Essumang, C. K. Adokoh, L. Boamponsem

**Affiliations:** Environmental Research Group, Department of Chemistry, University of Cape Coast, Cape Coast, Ghana

**Keywords:** neutron activation analysis, platinum group metals, Sarotherodon melanotheron, Chonophorus lateristriga, Macrobrachium vollenhovenii, Crassostrea tulipa

## Abstract

The use of some biota as bioindicators of heavy metal pollution has been demonstrated as particularly adequate due to their capacity of bioconcentration. This study evaluated the levels of platinum group metals (PGMs) in some selected species along the coastal belt of Ghana, using the neutron activation analysis (NAA) method. The result was processed to evaluate pollution indices in order to map the distribution of the metals in those species in the lagoons and estuaries along the costal belt of Ghana. The analysis showed significant levels of all PGMs in blackchin tilapia (*Sarotherodon melanotheron* Cichlidae), brown goby (*Chonophorus lateristriga* Gobiidae), shrimp (*Macrobrachium vollenhovenii* Palaemonidae), and mangrove oysters (*Crassostrea tulipa* Ostreidae) in the lagoons and river Pra estuary. However, the oysters showed an elevated mean concentration of 0.13 μ/g (dry weight) Pd. From the pollution indices, most of the sampling sites registered mean contamination factor (CF) values between 1.20 and 3.00 for Pt, Pd, and Rh. The pollution load index (PLI) conducted also gave an average pollution index between 0.79 and 2.37, indicating progressive contamination levels. The results revealed that anthropogenic sources, industrial and hospital effluent, etc., together with vehicular emissions, could be the contributing factors to the deposition of PGMs along the Ghanaian coast.

### INTRODUCTION

Heavy metals play a major role among pollutants of environmental concern and many of these metals, such as lead (Pb) or cadmium (Cd), are well studied in respect of their effects on living organisms[1]. In contrast, information on metals, such as platinum (Pt), palladium (Pd), and rhodium (Rh), referred to as platinum group metals (PGMs), which have been predominantly released in the last few decades as a consequence of anthropogenic activities, are scarce[2].

Mining, industries, hospitals, and other medical institutions are known to release PGMs into the environment, especially Pt, because it is used in anticancer drugs and dentistry[2,3]. The greatest part of PGM emissions, however, can be attributed to automobile traffic due to the use of PGMs in catalytic converters for purification of exhaust fumes from hydrocarbons, carbon monoxide, and nitrogen oxides[2,3,4].

Concentrations of PGMs have been found to be increasing in dust and soils along highways, and are transported into aquatic habitats through surface runoff, where they accumulate in the sediments of streams and in the tissues of aquatic organisms[5,6,7]. As a result, PGMs are becoming an emerging class of contaminants with potential human and environmental health implications, due to their suspected mutagenic and carcinogenic activities[8]. The elevated PGM levels in the environment have been largely linked to the introduction of automobile catalysts[4]. Since they occur naturally in very low concentrations[9,10], as such, they may have the potential to serve as an excellent indicator of highway and transportation impacts to aquatic and terrestrial ecosystems.

Marine organisms take up metals from their environment, which then accumulate in their tissues. Analysis of trace metals in tissues of marine organisms is a tool employed in marine pollution studies[11]. Measurement of PGMs of ecological, climatic, and anthropogenic changes underpins the formulation of effective management strategies for sustainable use and protection of the marine environment. Studies on PGMs in Ghana may serve as an early warning sign that has a direct bearing on vehicular flow. The levels of PGMs in *Sarotherodon melanotheron, Chonophorus*: *lateristriga, Macrobrachium vollenhovenii*, and *Crassostrea tulipa* have never been evaluated in Ghana, even though most developed countries do have the necessary data[5,6,7]. Interestingly, these species are consumed in Ghana, which implies that the continuous consumption of these species may not exempt the consumers from all the possible health effects associated with PGMs[12].

This study measured the extent of Pt, Pd, and Rh contamination in some species — blackchin tilapia (*Sarotherodon melanotheron* Cichlidae), brown goby (*Chonophorus*: *lateristriga*. Gobiidae), shrimp (*Macrobrachium vollenhovenii* Palaemonidae), and mangrove oysters *(Crassostrea tulipa* Ostreidae) — found in estuaries and lagoons along the coastal belt of Ghana. To determine the extent of PGM pollution in the estuaries, we compared the results to that of the U.K. permissible levels of heavy metal concentrations and also assessed their pollution status and their possible sources through the use of statistical/data treatment tools, such as contamination factor (CF) and pollution load index (PLI).

### MATERIALS AND METHODS OF ANALYSIS

#### Study Areas

These are the Pra Estuary at Shama (Western Region); Benya Lagoon at Elmina, Fosu Lagoon in Cape Coast, and Narkwa Lagoon at Narkwa (all in Central region); Sakumo 2 Lagoon near Tama (Greater Accra); and the Volta Estuary at Anyanuin and Keta Lagoon (both in the Volta Region). Each of these water bodies plays a significant role as far as fishery activities are concerned in Ghana. The habitats lie between latitudes 5° and 6°N and longitudes 1° 7’ E and 1° 45’ W, Fig. 1.

#### Sample Collection, Preparation, and Storage

Approximately 1 kg of the tilapia (*Sarotherodon melanotheron*) and brown goby (*Chonophorus lateristriga*) was collected from each habitat, where they occurred, using cast nets. The tilapia specimens were scaled and gutted, but the goby was not. (This is the how the respective fish species are treated before they are cooked and eaten.) Each species was dried in an oven at 40°C to a constant weight.

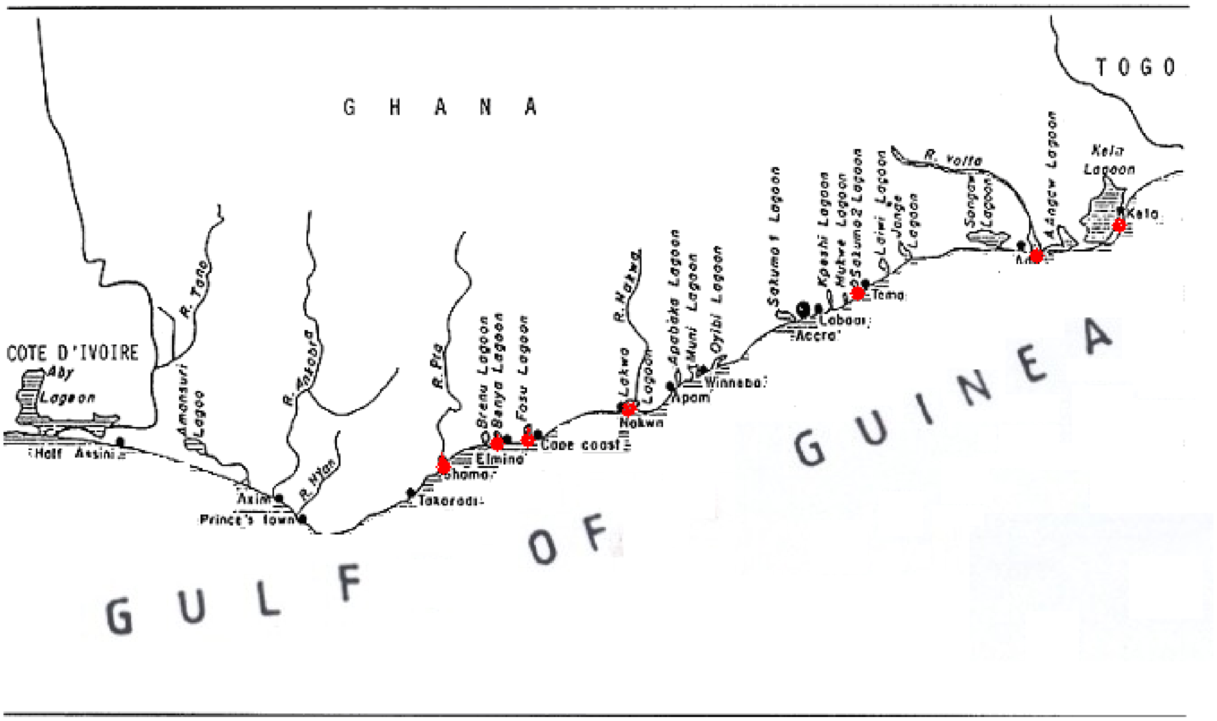

**FIGURE 1**. A map of Southern Ghana showing the sampling sites (in red).

Approximately 500 g of each was weighed and stored in well-labeled, white polyethylene bags and later sent to the Chemistry Department of the Ghana Atomic Energy Commission (GAEC) in Accra, for analysis.

About 1 kg of the shrimp (*Macrobrachium vollenhovenii*) was obtained from fishermen operating in the habitat where they had been caught with special traps. The exoskeleton, the head, appendages, and tail fan were removed and treated the same way as was done to tilapia and brown goby.

Samples of the oysters (*Crassostrea tulipa*) were collected from two stations in each habitat where they are available. They were washed clean, shucked, and treated likewise.

Sampling was done four times in the two main seasons (dry and wet seasons; twice in each season) within the year; January to April for the dry season and June to July for the wet season.

The oven-dried fish samples (tilapia, goby, shrimp, and mangrove oyster) were homogenized for further analysis. The muscle of the oysters (oven-dried) was also homogenized for further analysis. About 200 mg dry weight of each part of the fish samples were weighed by a Mettler Electronic Balance AE 163-BDH into a clean polyethylene film. The films were wrapped and heat sealed. The samples were packed into 7-mL volume rabbit capsules for irradiation. Two subsamples of each sample from all the sampling points and an IAEA standard reference material SARM 7 (certified standard for Pt, Pd, and Rh) were prepared and treated in the same manner as the samples[13].

### Sample Analysis

The determination of the trace PGMs was done by use of neutron activation analysis (NAA) using thermal neutron from a low-flux Am-Be radioisotope. Theoretically, NAA is based on the measurement of characteristic gamma rays from a radionuclide formed from the specific neutron reaction, which can be used to measure the amount of element using the usual radioactive decay law[14,15].

### Irradiation Source

The irradiation source was a 20-Ci Am-Be radioactive neutron source. It was cylindrically shaped and fixed in a holder at the center of a fiberglass tank, filled with deionized water. The deionized water served dual purposes, as a moderator and also as an absorber of neutrons. Extra shielding was provided by concrete blocks arranged around the tank. Transfer of sample to and from the neutron source was by means of a flexo-rabbit pneumatic transfer system operating under a pressure of 15 psi, given a sample transfer time of 1.3 sec[16]. The thermal neutron flux at the irradiation site was $1.124 \times 10^5$ ns$^{-1}$ cm$^{-2}$.

### Sample Irradiation

Each of the samples was sent by the pneumatic transfer system into the Am-Be source for irradiation. The irradiation schemes were chosen so as to take into account the half-lives of the radionuclides. In this regard, 1 h was chosen for all the samples because all the metals in question are medium-lived. At the end of each irradiation, the sample was returned for counting. Taking interference into consideration, samples were irradiated for 1 h and left overnight (16 h) for the decay process to take place and again after 5 days[16]. This is to allow optimized detection of $^{109}$Pd at 88 keV, Pt as $^{199}$Au at 158 keV, and $^{104m}$Rh at 51 keV. The samples were then counted the next day for 600 sec and intensities saved for further analysis. In order to avoid interference, preconcentration (fire assay) was employed, whereby samples were irradiated directly for gamma-ray spectroscopy[17]. In addition, as nuclear interference is primary, it is envisaged that interference will be negligible since the interference elements are not the matrix and the aiming elements are present in trace amounts[18].

### Data Processing

The detector type used for the counting of signals was an ENERTEC High Germanium (HPGe) detector of 3000 (+ve) bias and a resolution of 2.55 keV for 1332 KeV photo peak of Co-60. The signals from the detector were passed through the spectroscopy amplifier, and then accumulated by the Canberra Multi-Channel Analyzer (MCA) for a preset time. The spectra from the MCA were transferred to a DEC 350 microcomputer for analysis using Gamma spectrum analysis software (Ortec multichannel buffer [MCB]). A Microsoft Window–based software, MAESTRO, was used for spectrum analysis (i.e., qualitative and quantitative analysis)[15]. This software identifies the various photo peaks, estimates and works out the areas under them[14,17,19,20,21].

### Validation of Analytical Method

Validation of the analytical procedure was undertaken by irradiating an IAEA standard reference material Pt ore (SARM 7) and counting under identical experimental conditions. The analytical values of the reference material obtained from this study were compared with the recommended values (in ppm).

### Data Treatment

To know the pollution status of the studied environment, the PLIs and CF were computed using Microsoft Excel 2007. The mean concentrations and standard deviations for the biota species data were determined using SPSS version 16 software. According to Tomlinson et al.[22], indices enable quality of the environment to be easily understood by the nonspecialist.

### CF and PLI

The water pollution status of the study area was quantified using the CF approach[16,23]:

$$CF = Cs/Cc$$

where Cs = the average concentration of element in the samples and Cc = the average concentration of element in the standards, or control, or an unpolluted area. In this study, average concentrations of 0.026, 0.097, and 0.003 μg/g for Pt, Pd, and Rh, respectively, from Narkwa Lagoon (unpolluted area) were used since it is far away from heavy traffic areas, and there are no industries or hospitals located at this site. The only human activity at this site is fishing[15].

According to Tomlinson et al.[22] and Cabrera et al.[24], PLI is an empirical index that provides a simple, comparative means for assessing the level of heavy metal pollution. PLI was used to find the mutual pollution effect on each lagoon by the different metals in sediments and water. The PLI values were determined as the nth Root of the product of the n CF[15,25]:

$$\text{PLI sampling site} = 3\sqrt{}\text{CFPt} \times \text{CFPd} \times \text{CFRh}$$

where CF = contamination factor or pollution index factor (PIF). According to Tomlinson et al.[22], a PLI value of less than zero signifies an unpolluted area and value greater than zero shows a progressive deterioration of the environment. A PLI value of 1 implies that only baseline levels of pollutant are present.

The PLI and CF ranges, pollution grades, and intensities are given in Tables 1 and 2. The CF and PLI values of the elements in the analyzed biota from all the sampling points in the study area are given in Table 5.

**TABLE 1**
**CF Ranges and Their Designated Pollution Grade and Intensity**

| CF | Grade | Intensity |
|---|---|---|
| <1.2 | I | Unpolluted area |
| 1.2–2 | II | Lightly polluted area |
| 2–3 | III | Medium polluted area |
| >3 | IV | Heavily polluted area |

Source: Nyarko et al.[16].

**TABLE 2**
**PLIs and Their Pollution Grade and Intensity**

| PLI Value | Grade | Intensity |
|---|---|---|
| <0 | I | Unpolluted |
| 1 | II | Only baseline levels of pollutant present |
| >100 | III | Progressive deterioration of the environment |

Sources: Tomlinson et al.[22], Nyarko et al.[26], Angulo[27].

## RESULTS

### Quality Assurance

The accuracy and precision of the analytical technique (INAA) was assessed by simultaneous activation of certified reference material SARM 7 (Pt ore) prepared by the National Institute for Metallurgy and distributed by the South African Bureau of Standards. In fact, there were some challenges as there was no biological reference material available that had been certified for PGM. Also, there was a difficulty in using an intermethod/interlaboratory comparison and, as a result, an available Pt ore certified reference material was used to validate the methodology. Table 3 shows the analytical results obtained at GHARR-1 laboratory for the reference material compared with the recommended values. The values compared favorably well with the recommended values for Pt, Pd, and Rh, with bias less than 6%. The precision was calculated as a percentage relative standard deviation (%RSD) of three replicate samples of the prepared standard, and was found to be less than 5% with percentage recovery of about 98%.

**TABLE 3**
**Result of the Quality Control Analysis**

| PGMs (µg/g) | Standard Concentration SARM 7 | Results Obtained after NAA Analysis of the Standards | | | Mean | % Recovery |
|---|---|---|---|---|---|---|
| | | Std. 1 | Std. 2 | Std. 3 | | |
| Pt | 3.74 ± 0.045 | 3.65 ± 0.55 | 3.70 ± 0.56 | 3.71 ± 0.56 | 3.69 ± 0.56 | 98.6 |
| Pd | 1.53 ± 0.032 | 1.49 ± 0.22 | 1.50 ± 0.23 | 1.50 ± 0.23 | 1.50 ± 0.23 | 98.0 |
| Rh | 0.24 ± .013 | 0.24 ± 0.04 | 0.22 ± 0.033 | 0.25 ± 0.04 | 0.24 ± 0.04 | 98.95 |

### PGM Concentration in Biota

The result of the analysis of PGMs by NAA in the tilapia, goby, shrimp, and oysters from the Pra and Volta Estuaries and the Benya, Fosu, Narkwa, Sakumono 2, and Keta Lagoons showed significantly elevated levels of the metals in the selected species compare to the background concentration. The concentrations of PGMs with their mean values are tabulated in Tables 1–7 in the Appendix. The tables show the mean concentrations of PGMs in composite samples of biota sampled for four consecutive sampling occasions (two times each in the dry and wet seasons). The metal concentrations in the different biota generally seem to be highest in the samples from Benya Lagoon followed by Pra, Fosu, Volta, Narkwa, Sakumono 2, and Keta, in that order. These areas, except Narkwa, are all experiencing dense traffic or high vehicular activities, which might have contributed to the elevated PGM levels. Higher concentrations of the metals were recorded mostly in the dry season for all three metals at almost all the sampling sites. The only exception is the oyster samples from Benya Lagoon, recording a higher value of 0.161 ± 0.024 µg/g (dry weight) for Pd, and Keta Lagoon also having 0.128 ± 0.019 (dw) for Pt in the wet season. The next highest concentration was also at Benya Lagoon in the tilapia sample in the dry season (0.146 ± 0.022 µg/g dry weight) Pd (Table 4). The higher mean concentration was 0.131 µg/g Pd (oyster) followed by tilapia (0.099 µg/g Pt) at Pra Estuary. Similar PGM concentrations (0.040–0.481 µg/g for Pd, 0.239–0.946 µg/g for Pt, and 0.011–0.037 µg/g for Rh) in dolphins (*Stenella* sp.) along the Ghanaian coastline have been reported by Essumang[28].

**TABLE 4**
**Mean Concentration (µg/g) Dry Weight of PGMs Measured from Biota Samples along the Coastal Belt of Ghana (± = SE)**

| Sampling Sites | Biota | Seasons | PGMs (µg/g) | | |
|---|---|---|---|---|---|
| | | | Pt | Pd | Rh |
| Pra Estuary (1) | Tilapia | Dry | 0.113 ± 0.016 | 0.061 ± 0.017 | 0.002 ± 0.001 |
| | | Wet | 0.085 ± 0.013 | 0.012 ± 0.002 | 0.001 ± 0.000 |
| | | **Mean** | **0.099** | **0.0365** | **0.0015** |
| | | SD | 0.019 | 0.035 | 0.0007 |
| | Shrimp | Dry | 0.100 ± 0.015 | 0.022 ± 0.003 | 0.0015 ± 0.000 |
| | | Wet | 0.051 ± 0.008 | 0.130 ± 0.019 | 0.001 ± 0.000 |
| | | **Mean** | **0.076** | **0.076** | **0.001** |
| | | SD | 0.035 | 0.076 | 0.000 |
| Benya Lagoon (2) | Tilapia | Dry | 0.060 ± 0.017 | 0.059 ± 0.009 | 0.009 ± 0.001 |
| | | Wet | 0.014 ± 0.003 | 0.146 ± 0.022 | 0.002 ± 0.000 |
| | | **Mean** | **0.037** | **0.103** | **0.006** |
| | | SD | 0.033 | 0.062 | 0.005 |
| | Brown Goby | Dry | 0.017 ± 0.004 | 0.071 ± 0.011 | 0.002 ± 0.000 |
| | | Wet | 0.005 ± 0.000 | 0.014 ± 0.002 | 0.0003 ± 0.000 |
| | | **Mean** | **0.011** | **0.0425** | **0.0012** |
| | | SD | 0.008 | 0.040 | 0.0012 |
| | Oyster | Dry | 0.006 ± 0.001 | 0.100 ± 0.015 | 0.003 ± 0.001 |
| | | Wet | 0.082 ± 0.012 | 0.161 ± 0.024 | 0.003 ± 0.001 |
| | | **Mean** | **0.044** | **0.131** | **0.003** |
| | | SD | 0.054 | 0.043 | 0.000 |
| Fosu Lagoon (3) | Tilapia | Dry | 0.083 ± 0.012 | 0.093 ± 0.014 | 0.002 ± 0.000 |
| | | Wet | 0.041 ± 0.011 | 0.054 ± 0.016 | 0.002 ± 0.000 |
| | | **Mean** | **0.079** | **0.070** | **0.002** |
| | | SD | 0.005 | 0.033 | 0.000 |
| Narkwa Lagoon (4) | Tilapia | Dry | 0.019 ± 0.003 | 0.005 ± 0.000 | 0.003 ± 0.000 |
| | | Wet | 0.006 ± 0.001 | 0.107 ± 0.004 | 0.001 ± 0.000 |
| | | **Mean** | **0.0125** | **0.056** | **0.002** |
| | | SD | 0.009 | 0.072 | 0.001 |
| | Oyster | Dry | 0.025 ± 0.006 | 0.105 ± 0.016 | 0.004 ± 0.001 |
| | | Wet | 0.033 ± 0.005 | 0.084 ± 0.013 | 0.001 ± 0.000 |
| | | **Mean** | **0.029** | **0.095** | **0.0025** |
| | | SD | 0.006 | 0.015 | 0.002 |
| Sakumono 2 Lagoon (5) | Tilapia | Dry | 0.046 ± 0.013 | 0.019 ± 0.003 | 0.001 ± 0.000 |
| | | Wet | 0.027 ± 0.007 | 0.103 ± 0.015 | 0.003 ± 0.001 |
| | | **Mean** | **0.0365** | **0.061** | **0.002** |
| | | SD | 0.034 | 0.059 | 0.001 |
| Volta Estuary (6) | Oyster | Dry | 0.088 ± 0.013 | 0.051 ± 0.007 | 0.0035 ± 0.000 |
| | | Wet | 0.040 ± 0.006 | 0.0596 ± 0.009 | 0.0011 ± 0.000 |
| | | **Mean** | **0.064** | **0.055** | **0.0023** |
| | | SD | 0.034 | 0.006 | 0.002 |
| Keta Lagoon (7) | Tilapia | Dry | 0.049 ± 0.007 | 0.027 ± 0.007 | 0.0015 ± 0.000 |
| | | Wet | 0.128 ± 0.019 | 0.049 ± 0.007 | 0.003 ± 0.001 |
| | | **Mean** | **0.089** | **0.038** | **0.0023** |
| | | SD | 0.056 | 0.016 | 0.001 |
| | Brown Goby | Dry | 0.016 ± 0.004 | nd | 0.0015 ± 0.0000 |
| | | Wet | 0.005 | nd | 0.001 |
| | | **Mean** | **0.0105** | **nd** | **0.001** |
| | | SD | 0.000 | 0.000 | 0.000 |

### The Levels of PGM in Fish and Shellfish

In general, elevated levels of the PGMs were observed in all the studied fish samples; however, higher mean concentrations of PGMs were found in the blackchin tilapia compared to the brown goby (Table 4). The Pd concentration was the highest among the PGMs, with tilapia recording 0.146 ± 0.022 μg/g dry weight (Table 4), followed by Pt (0.128 ± 0.019 μg/g dry weight), and the least being Rh (0.009 ± 0.001 μg/g dry weight) all in tilapia. The highest concentration of PGMs measured in brown goby is 0.071 ± 0.011 μg/g dry weight (Table 4).

In the case of shellfish, mean concentrations of PGMs in the shrimp did not differ significantly, as compared with that of fish. The level of accumulation was almost the same as in the fish, but the order of the levels is as follows: Pd (0.130 ± 0.019 μg/g) > Pt (0.100 ± 0.015 μg/g) > Rh (0.0015 ± 0.000 μg/g) (dry weight) (Table 4). The concentration pattern in oysters changed drastically as the mean concentration increased by about 56% as compared to even its classmate crustacean shrimps (see Appendix Table 1). The highest concentration recorded in the mangrove oyster was for Pd (0.161 ± 0.024 μg/g) (dry weight) (Table 4).

### CFs

The variation of CF across the sampling points is shown in Table 5. Tilapia from Keta Lagoon (sampling point 7 [SP7]) sampled during the wet season had the highest Pt CF of 4.93, followed by dry season tilapia at the Benya Lagoon sampling point of 4.43 for Pd. Keta (SP7) recorded the lowest value of below LOD (limit of determination) for Pd in both the dry and wet season for the goby sample. The second highest CF (3.20) of Rh occurred at Benya (SP2) in the tilapia taken in the dry season, and the lowest CF (0.00) was recorded in wet season tilapia at Narkwa (SP4) and Keta (SP7). Among the metals, Pd recorded the least CF values, ranging from LOD to 2.09 (Table 5).

The highest mean (1.37) and the lowest mean (0.79) PLIs occurred at the sampling sites in Benya and Keta Lagoons, respectively. The sequence of PLI values of the studied sampling sites is as follows: Benya Lagoon (1.37) > Pra Estuary (1.27) >Fosu Lagoon (1.20) >Volta Estuary (1.12) > Sakumono 2 Lagoon (0.98) > Narkwa Lagoon (0.93) > Keta Lagoon (0.79) (Table 5).

## DISCUSSION

### PGM Levels

Elevated PGM concentrations were generally recorded in biota from Benya Lagoon compared to other sites. Pd was the most highly concentrated metal, with a value of 0.161 ± 0.024 μg/g in the oyster, followed by Pt with 0.113 ± 0.016 μg/g, while the least concentration was Rh (0.009 ± 0.001 μg/g) in the tilapia from the Benya Lagoon and Pra Estuary in the case of Pt. The concentration was seen to be slightly lower as compared to recent findings by Essumang et al.[13], which indicated accumulation among some of the species involved. All the lagoons and estuaries along highways showed elevated levels. This is in line with the findings by Essumang et al.[29], who reported high concentrations of PGMs in road dust, sediments from the river bank, sediments 3 m from the river bank, sediments from the waterbed, and water samples taken from the Pra Estuary and its surroundings. The highest concentrations in road dust (0.537 ± 0.081 μg/g of Pd and 0.189 ± 0.028 μg/g for Pt) were found on the bridge (old and new) and its immediate surroundings, which lie across the Pra Estuary[29]. In addition, Pd was found to have the highest concentration in almost all the fish samples used for that research[13]. In this present research, the same trend has been observed and suggests that Pd seems to be the most mobile element among the PGMs, with a mobility gradient of Pd > Pt ≥ Rh, comparing their highest values to their earth's crust background levels (0.005, 0.005, and 0.0002 ppm for Pt, Pd, and Rh, respectively)[30,31]. The same result

**TABLE 5**
**Biota and Their Pollution Index Factors and Contamination Factors**

| Sampling Site | Biota | Season | CF | | | PLI | Average PLI |
|---|---|---|---|---|---|---|---|
| | | | Pt | Pd | Rh | | |
| Pra Estuary | Tilapia | Dry | 4.16 | 1.19 | 1.13 | 1.78 | |
| | | Wet | 3.26 | 0.12 | 0.47 | 1.50 | |
| | | **Mean** | **3.71** | **0.66** | **0.80** | **1.25** | |
| | | Dry | 3.86 | 0.23 | 0.80 | 0.89 | |
| | | Wet | 2.10 | 2.09 | 0.17 | 0.90 | |
| | Shrimp | **Mean** | **2.98** | **1.16** | **0.48** | **1.19** | **1.27** |
| Benya Lagoon | Tilapia | Dry | 4.43 | 0.61 | 3.20 | 2.06 | |
| | | Wet | 0.87 | 1.50 | 0.80 | 1.01 | |
| | | **Mean** | **2.65** | **1.06** | **2.00** | **1.78** | |
| | Goby | Dry | 1.15 | 0.74 | 1.00 | 0.94 | |
| | | Wet | 0 | 0.14 | 0.10 | 0.12 | |
| | | **Mean** | **1.15** | **0.44** | **0.55** | **0.65** | |
| | Oyster | Dry | 0.22 | 1.04 | 1.40 | 0.68 | |
| | | Wet | 3.15 | 1.66 | 1.67 | 2.06 | |
| | | **Mean** | **1.68** | **1.35** | **1.53** | **1.52** | **1.37** |
| Fosu Lagoon | Tilapia | Dry | 3.18 | 0.96 | 0.93 | 1.42 | |
| | | Wet | 2.93 | 0.48 | 0.67 | 0.98 | |
| | | **Mean** | **3.06** | **0.72** | **0.80** | **1.21** | **1.20** |
| Narkwa Lagoon | Tilapia | Dry | 0.74 | 0.00 | 1.13 | 0.91 | |
| | | Wet | 0.26 | 1.46 | 0.27 | 0.47 | |
| | | **Mean** | **0.50** | **1.46** | **0.70** | **0.80** | |
| | Oyster | Dry | 1.72 | 1.08 | 1.20 | 1.30 | |
| | | Wet | 1.27 | 0.86 | 0.00 | 1.03 | |
| | | **Mean** | **1.49** | **0.97** | **1.20** | **1.20** | **0.93** |
| Sakumono 2 Lagoon | Tilapia | Dry | 3.32 | 0.19 | 0.40 | 0.64 | |
| | | Wet | 1.88 | 1.06 | 1.13 | 1.31 | |
| | | **Mean** | **2.60** | **0.63** | **0.77** | **1.08** | **0.98** |
| Volta Estuary | Oyster | Dry | 3.38 | 0.52 | 2.00 | 1.52 | |
| | | Wet | 1.55 | 0.61 | 0.40 | 0.72 | |
| | | **Mean** | **2.47** | **0.57** | **1.2** | **1.19** | **1.12** |
| Keta Lagoon | Tilapia | Dry | 1.88 | 0.50 | 0.60 | 0.83 | |
| | | Wet | 4.93 | 0.50 | 1.27 | 1.46 | |
| | | **Mean** | **3.41** | **0.50** | **0.93** | **1.17** | |
| | Goby | Dry | 1.05 | 0.00 | 0.69 | 0.85 | |
| | | Wet | 0.00 | 0.00 | 0.00 | 0.00 | |
| | | **Mean** | **1.05** | **0.00** | **0.69** | **0.85** | **0.79** |

has been reported by Sures et al.[5] in a study of a road dust that revealed very high levels of Pd. The increased proportion of Pd in the road dust may be due to changes in the composition of metal mixtures in catalytic converters used for automobile exhaust purification[5,32]. The portion of Pd in catalysts has increased to approximately 96%, showing a dominant use of Pd in catalytic converters in recent years[33].

### The Levels of PGMs in Fish and Shellfish

In general, elevated levels of the PGMs were observed in all the studied fish samples and this observation is similar to a study by Essumang[28]. This was observed for almost all the species used in this research (see Appendix tables).

Zimmermann et al.[7] also reported that crustaceans incorporate heavy metals into their exoskeletons (shells). The high concentration of PGMs found in the oysters has been attributed to the fact that they have intimate contact with the sediments, as they are bottom-dwelling animals. They are also static or slow moving organisms, i.e., their intimate contact with sediment may contribute immensely to their elevated PGM levels. According to Zimmermann et al.[7], bivalves have a high capacity for accumulating PGMs in their aquatic biosphere and, so, the use of oysters to monitor noble metals in the aquatic ecosystems is very important.

Research with terrestrial plants and animals has shown that the transfer of PGMs from contaminated soils into plants and animals decreases in the order of Pd > Pt ≥ Rh[32] and that Pd is bound to a high variety of plant proteins[34]. Thus, Pd seems to be the most environmentally mobile and biologically available metal among the PGMs. Our research results compared to mean U.K. dietary intake (Pt [0.2 μg/g/day], Pd [1.0 μg/g/day], and Rh [0.2 μg/g/day]) were found to be slightly lower[2,35]. These levels compared with previous work done by Essumang et al.[13], and Essumang[28] confirms an accumulation of these metals in the fish species in water bodies close to highways. The elevated Pt concentrations obtained from studies in Ghana by Kylander et al.[36] compared to levels in roadside soils from Europe and the U.S. were unexpected due to the prolonged use of catalysts in vehicles in Europe and the U.S.

This might be an indicator that gold mining in Ghana may have contributed to the elevated PGMs in the Ghanaian environment. Ghana has a very long history of gold mining, and Au and PGMs are commonly associated in terrestrial rocks. However, there has not been any study on the levels PGMs from the very large debris from pond failures and transport of tailings enriched with PGMs.

In short, Ghana is covered by the Paleoprotoerozoic rocks of the Birimian Supergroup and the overlying clastic sedimentary Tarkwaian group[37]. As a result of a series of erosional events, significant portions of these rocks have been redeposited as placer formations in a number of streams and channels. Placer gold deposits, which are also referred to as “alluvial gold”, are found in a majority of rivers draining Birimian rocks. Large deposits of placer gold also occur along the terraces, floodplains, channels, and riverbeds of the Offin, Pra, Ankobra, Birim, and Tano Rivers, where large Birimian and Tarkwaian gold deposits have experienced several episodes of erosion and subsequent deposition[38]. All these water bodies deposit their debris into the Gulf of Guinea; hence, the possibility of elevated PGM levels.

The effects of PGMs on animals and the environment have not been studied extensively in Ghana. Evidence indicates that PGMs, particularly Pd and Pt, are transported to biological materials by binding to sulfur-rich low-molecular-weight species in plant roots[4]. The metals tend to accumulate in the roots of plants after uptake from the soil and/or in humans from eating contaminated foods. Pt and its compounds have a wide spectrum of toxicity, ranging from relatively low toxicity to genotoxic/cytoxic effect and sensitization reactions. These are associated with the Pt salts and its complexes. Its effect on humans is not yet fully known. However, it is believed that many microorganisms convert Pt in soils to very harmful compounds that could cause several health problems, such as cancer, allergic reactions, DNA alterations, and mucous membrane destruction[39].

### Pollution Survey Analysis

Analysis of CFs and PLIs indicates how vehicular activities are contributing to PGM pollution in the environment. Only few Pt CF values were found to be greater than 3 (Table 5), which showed how the sampling points are gradually being polluted through anthropogenic sources. Results in Table 5 show that most of the sampling sites recorded CF values between 1.2 and 2.0, meaning that they are slightly polluted by the PGMs through anthropogenic sources[16]. Few others also showed medium polluted areas since their CF values where between 2 and 3 (CF values recorded in shrimp at the Pra Estuary for Pt /Pd and mean values from Benya Lagoon in tilapia for Pt and Rh, Table 5). Also, almost all of the average PLI values obtained were markedly less than 100 (i.e., 0.79–2.37), indicating low contamination with Pt, Pd, and Rh (cf. [27]). The PLI values recorded at Benya and Fosu Lagoons and the Pra and Volta Estuaries were more than 1, signifying a baseline level of pollution. The PGM concentrations in all the sampling sites are accumulating from high vehicular activities, especially at the Benya Lagoon sampling site where there was heavy traffic[4].

Using the data generated from the concentrations in biota, sampling sites exposed to higher amounts of the pollutants were identified. Results in Table 5 revealed that the sampling sites (Benya, Fosu, Pra, and Volta water bodies) recorded mean PLI values greater than 1, while Sakumono 2, Narkwa, and Keta Lagoons had values approximately equal to 1. This suggests that the heavy vehicular movement areas had the highest PLI values of 1.37, 1.27, 1.20, and 1.12 for Benya Lagoon, Pra Estuary, Fosu Lagoon, and Volta Estuary, respectively (Table 5). All these sites lie close to major highways.

### CONCLUSION

The results of this work have indicated that vehicular activities on the Ghanaian highways emit PGMs along the road. Some of these PGMs are discharged into water bodies (the Pra and Volta Estuaries, and Benya Lagoon at Elmina, Fosu Lagoon in Cape Coast, Sakumono 2 Lagoon along the Accra – Tama road, and Keta Lagoon in Keta) by runoff.

The CF and PLI analyses conducted have revealed that the seven water bodies of the study areas have elevated levels of Pd, Pt, and Rh in excess of the background values. This indicates that the abundant nature of these elements in the atmosphere of the study area has reached polluted status on the pollution scale.

The presence of Pd and Pt should be of great interest to researchers, as the portion of Pd in the metal mixtures used in catalytic converters has increased over time. In addition, Pt is of a particular concern as it has a known mutagenic and toxic effect, even at exceedingly low concentrations in water bodies (affecting ecosystems). It is envisaged that the results of this study will enrich the discussion and understanding of the effects of vehicular activities on the environment, as well as the health implications on the people.

### ACKNOWLEDGMENT

The authors wish to express their sincere appreciation to the staff of Ghana Atomic Energy Commission at Kwabenya - Accra, for their kind assistance in the analysis of the samples. Sincere thanks also go to the entire laboratory staff of the Chemistry Department, University of Cape Coast for their support. Finally, we wish to thank the government of Ghana for financial assistance.

## APPENDIX 1

### Levels of PGMs Measured from Biota Samples along the Coastal Belt of Ghana

**TABLE 1**

| Sampling Sites | Biota | Seasons | PGMs (µg/g Dry Weight) | | |
|---|---|---|---|---|---|
| | | | Pt | Pd | Rh |
| Pra Estuary | Tilapia | Dry 1 | 0.108 ± 0.016 | 0.005 ± 0.000 | 0.003 ± 0.001 |
| | | Dry 2 | 0.005 ± 0.000 | 0.116 ± 0.017 | 0.001 ± 0.000 |
| | | **Mean** | **0.113 ± 0.016** | **0.061 ± 0.017** | **0.002 ± 0.001** |
| | | Wet 1 | 0.101 ± 0.015 | 0.0134 ± 0.002 | 0.001 ± 0.000 |
| | | Wet 2 | 0.069 ± 0.0104 | 0.0106 ± 0.002 | 0.001 ± 0.000 |
| | | **Mean** | **0.085 ± 0.013** | **0.012 ± 0.002** | **0.001 ± 0.000** |
| | Shrimp | Dry 1 | 0.1626 ± 0.024 | 0.009 ± 0.006 | 0.001 ± 0.000 |
| | | Dry 2 | 0.038 ± 0.006 | 0.036 ± 0.005 | 0.002 ± 0.000 |
| | | **Mean** | **0.100 ± 0.015** | **0.022 ± 0.003** | **0.0015 ± 0.000** |
| | | Wet 1 | 0.067 ± 0.01 | 0.213 ± 0.032 | 0.001 ± 0.000 |
| | | Wet 2 | 0.042 ± 0.006 | 0.047 ± 0.007 | 0.002 ± 0.000 |
| | | **Mean** | **0.051 ± 0.008** | **0.130 ± 0.019** | **0.0015 ± 0.000** |

**TABLE 2**

| Sampling Sites | Biota | Seasons | PGMs (µg/g Dry Weight) | | |
|---|---|---|---|---|---|
| | | | Pt | Pd | Rh |
| Fosu Lagoon | Tilapia | Dry 1 | 0.144 ± 0.022 | 0.147 ± 0.022 | 0.003 ± 0.000 |
| | | Dry 2 | 0.022 ± 0.003 | 0.038 ± 0.006 | 0.001 ± 0.000 |
| | | **Mean** | **0.083 ± 0.012** | **0.093 ± 0.014** | **0.002 ± 0.000** |
| | | Wet 1 | 0.005 ± 0.000 | 0.005 ± 0.000 | 0.002 ± 0.000 |
| | | Wet 2 | 0.076 ± 0.011 | 0.103 ± 0.016 | 0.004 ± 0.001 |
| | | **Mean** | **0.041 ± 0.011** | **0.054 ± 0.016** | **0.002 ± 0.000** |

**TABLE 3**

| Sampling Sites | Biota | Seasons | PGMs (µg/g Dry Weight) | | |
|---|---|---|---|---|---|
| | | | Pt | Pd | Rh |
| Benya Lagoon | Tilapia | Dry 1 | 0.005 ± 0.000 | 0.062 ± 0.009 | 0.010 ± 0.001 |
| | | Dry 2 | 0.115 ± 0.017 | 0.057 ± 0.009 | 0.009 ± 0.001 |
| | | **Mean** | **0.060 ± 0.017** | **0.059 ± 0.009** | **0.009 ± 0.001** |
| | | Wet 1 | 0.005 ± 0.000 | 0.224 ± 0.034 | 0.003 ± 0.000 |
| | | Wet 2 | 0.023 ± 0.003 | 0.067 ± 0.010 | 0.003 ± 0.000 |
| | | **Mean** | **0.014 ± 0.003** | **0.146 ± 0.022** | **0.002 ± 0.000** |
| | Goby | Dry 1 | 0.029 ± 0.004 | 0.022 ± 0.003 | 0.003 ± 0.000 |
| | | Dry 2 | 0.005 ± 0.000 | 0.1208 ± 0.018 | 0.001 ± 0.000 |
| | | **Mean** | **0.017 ± 0.004** | **0.071 ± 0.011** | **0.002 ± 0.000** |
| | | Wet 1 | nd | 0.015 ± 0.002 | 0.0002 ± 0.000 |
| | | Wet 2 | nd | 0.013 ± 0.000 | 0.0004 ± 0.001 |
| | | **Mean** | **nd** | **0.014 ± 0.002** | **0.0003 ± 0.000** |
| | Oyster | Dry 1 | 0.006 ± 0.001 | 0.082 ± 0.012 | 0.004 ± 0.001 |
| | | Dry 2 | 0.005 ± 0.000 | 0.1192 ± 0.018 | 0.001 ± 0.000 |
| | | **Mean** | **0.006 ± 0.001** | **0.100 ± 0.015** | **0.003 ± 0.001** |
| | | Wet 1 | 0.084 ± 0.013 | 0.166 ± 0.088 | 0.001 ± 0.000 |
| | | Wet 2 | 0.080 ± 0.013 | 0.156 ± 0.044 | 0.005 ± 0.001 |
| | | **Mean** | **0.082 ± 0.012** | **0.161 ± 0.024** | **0.003 ± 0.001** |

**TABLE 4**

| Sampling Sites | Biota | Seasons | PGMs (µg/g Dry Weight) | | |
|---|---|---|---|---|---|
| | | | Pt | Pd | Rh |
| Narkwa Lagoon | Tilapia | Dry 1 | 0.006 ± 0.001 | nd | 0.002 ± 0.000 |
| | | Dry 2 | 0.033 ± 0.005 | nd | 0.005 ± 0.001 |
| | | **Mean** | **0.019 ± 0.003** | **nd** | **0.003 ± 0.000** |
| | | Wet 1 | 0.005 ± | 0.109 ± 0.018 | 0.001 ± 0.000 |

| | | | | |
|---|---|---|---|---|
| | | 0.000 | | |
| | Wet 2 | 0.007 ± 0.001 | 0.104 ± 0.014 | 0.001 ± 0.000 |
| | **Mean** | **0.006 ± 0.001** | **0.107 ± 0.004** | **0.001 ± 0.000** |
| Oyster | Dry 1 | 0.005 ± 0.000 | 0.055 ± 0.008 | 0.001 ± 0.00 |
| | Dry 2 | 0.045 ± 0.006 | 0.154 ± 0.023 | 0.006 ± 0.001 |
| | **Mean** | **0.025 ± 0.006** | **0.105 ± 0.016** | **0.004 ± 0.001** |
| | Wet 1 | 0.006 ± 0.001 | 0.084 ± 0.013 | nd |
| | Wet 2 | 0.060 ± 0.009 | 0.005 ± 0.000 | nd |
| | **Mean** | **0.033 ± 0.005** | **0.045 ± 0.013** | **nd** |

**TABLE 5**

| Sampling Sites | Biota | Seasons | PGMs (µg/g Dry Weight) | | |
|---|---|---|---|---|---|
| | | | Pt | Pd | Rh |
| Sakumono 2 Lagoon | Tilapia | Dry 1 | 0.005 ± 0.000 | 0.036 ± 0.005 | 0.001 ± 0.000 |
| | | Dry 2 | 0.086 ± 0.013 | 0.002 ± 0.000 | 0.002 ± 0.000 |
| | | **Mean** | **0.046 ± 0.013** | **0.019 ± 0.003** | **0.001 ± 0.000** |
| | | Wet 1 | 0.005 ± 0.000 | 0.112 ± 0.017 | 0.001 ± 0.000 |
| | | Wet 2 | 0.049 ± 0.007 | 0.094 ± 0.014 | 0.005 ± 0.001 |
| | | **Mean** | **0.027 ± 0.007** | **0.103 ± 0.015** | **0.003 ± 0.001** |

**TABLE 6**

| Sampling Sites | Biota | Seasons | PGMs (µg/g Dry Weight) | | |
|---|---|---|---|---|---|
| | | | Pt | Pd | Rh |
| Volta Estuary | Oyster | Dry 1 | 0.038 ± 0.006 | 0.027 ± 0.004 | 0.006 ± 0.001 |
| | | Dry 2 | 0.138 ± 0.021 | 0.074 ± 0.011 | 0.001 ± 0.000 |
| | | **Mean** | **0.088 ± 0.013** | **0.051 ± 0.007** | **0.0035 ± 0.001** |
| | | Wet 1 | 0.050 ± 0.008 | 0.094 ± 0.014 | 0.001 ± 0.000 |
| | | Wet 2 | 0.030 ± 0.0046 | 0.025 ± 0.0036 | 0.0012 ± 0.000 |
| | | **Mean** | **0.040 ± 0.006** | **0.0596 ± 0.009** | **0.0011 ± 0.000** |

**TABLE 7**

| Sampling Sites | Biota | Seasons | PGMs (µg/g Dry Weight) | | |
|---|---|---|---|---|---|
| | | | Pt | Pd | Rh |
| Keta Lagoon | Tilapia | Dry 1 | 0.065 ± 0.010 | 0.005 ± 0.000 | 0.001 ± 0.000 |
| | | Dry 2 | 0.033 ± 0.005 | 0.049 ± 0.007 | 0.002 ± 0.000 |
| | | **Mean** | **0.049 ± 0.007** | **0.027 ± 0.007** | **0.0015 ± 0.000** |
| | | Wet 1 | 0.1634 ± 0.025 | 0.007 ± 0.001 | 0.001 ± 0.000 |
| | | Wet 2 | 0.0928 ± 0.014 | 0.090 ± 0.014 | 0.004 ± 0.001 |
| | | **Mean** | **0.128 ± 0.019** | **0.049 ± 0.007** | **0.003 ± 0.001** |
| | Goby | Dry 1 | 0.027 ± 0.004 | nd | 0.002 ± 0.000 |
| | | Dry 2 | 0.005 ± 0.000 | nd | 0.001 ± 0.000 |
| | | **Mean** | **0.016 ± 0.004** | **nd** | **0.0015 ± 0.000** |
| | | Wet 1 | nd | nd | nd |
| | | Wet 2 | nd | nd | nd |
| | | **Mean** | **nd** | **nd** | **nd** |
